# Data quality monitoring and performance metrics of a prospective, population-based observational study of maternal and newborn health in low resource settings

**DOI:** 10.1186/1742-4755-12-S2-S2

**Published:** 2015-06-08

**Authors:** Shivaprasad S Goudar, Kristen B Stolka, Marion Koso-Thomas, Narayan V Honnungar, Shivanand C Mastiholi, Umesh Y Ramadurg, Sangappa M Dhaded, Omrana Pasha, Archana Patel, Fabian Esamai, Elwyn Chomba, Ana Garces, Fernando Althabe, Waldemar A Carlo, Robert L Goldenberg, Patricia L Hibberd, Edward A Liechty, Nancy F Krebs, Michael K Hambidge, Janet L Moore, Dennis D Wallace, Richard J Derman, Kodkany S Bhalachandra, Carl L Bose

**Affiliations:** 1 Women’s and Children’s Health Research Unit, KLE University’s Jawaharlal Nehru Medical College, Belgaum, Karnataka, India; 2 RTI International, Durham, NC, USA; 3 Center for Research for Mothers and Children, Eunice Kennedy Shriver National Institute of Child Health and Human Development, Bethesda, Maryland, USA; 4 S Nijalingappa Medical College, Belgaum, Karnataka, India; 5 Department of Community Health Sciences, Aga Khan University, Pakistan; 6 Indira Gandhi Government Medical College and Lata Medical Research Foundation, Nagpur, Maharashtra, India; 7 University School of Medicine, Eldoret, Kenya; 8 University Teaching Hospital, University of Zambia, Lusaka, Zambia; 9 Department of Pediatrics, School of Medicine, San Carlos University, Guatemala City, Guatemala; 10 Institute for Clinical Effectiveness and Health Policy, Buenos Aires, Argentina; 11 Department of Pediatrics, Division of Neonatology, University of Alabama at Birmingham, School of Medicine, Birmingham, AL, USA; 12 Department of Obstetrics and Gynecology, Columbia University, New York, NY, USA; 13 Massachusetts General Hospital for Children, Boston, MA, USA; 14 Indiana University School of Medicine, Indianapolis, IA, USA; 15 University of Colorado School of Medicine, Denver, CO, USA; 16 Department of Pediatrics, Division of Neonatal-Perinatal Medicine, University of North Carolina School of Medicine, Chapel Hill, NC, USA; 17 Christiana Care Health Services, Newark, DE, USA

**Keywords:** data monitoring, data quality, maternal health, newborn health, perinatal registry, metrics, low-income countries

## Abstract

**Background:**

To describe quantitative data quality monitoring and performance metrics adopted by the Global Network’s (GN) Maternal Newborn Health Registry (MNHR), a maternal and perinatal population-based registry (MPPBR) based in low and middle income countries (LMICs).

**Methods:**

Ongoing prospective, population-based data on all pregnancy outcomes within defined geographical locations participating in the GN have been collected since 2008. Data quality metrics were defined and are implemented at the cluster, site and the central level to ensure data quality. Quantitative performance metrics are described for data collected between 2010 and 2013.

**Results:**

Delivery outcome rates over 95% illustrate that all sites are successful in following patients from pregnancy through delivery. Examples of specific performance metric reports illustrate how both the metrics and reporting process are used to identify cluster-level and site-level quality issues and illustrate how those metrics track over time. Other summary reports (e.g. the increasing proportion of measured birth weight compared to estimated and missing birth weight) illustrate how a site has improved quality over time.

**Conclusion:**

High quality MPPBRs such as the MNHR provide key information on pregnancy outcomes to local and international health officials where civil registration systems are lacking. The MNHR has measures in place to monitor data collection procedures and improve the quality of data collected. Sites have increasingly achieved acceptable values of performance metrics over time, indicating improvements in data quality, but the quality control program must continue to evolve to optimize the use of the MNHR to assess the impact of community interventions in research protocols in pregnancy and perinatal health.

**Trial registration number:**

NCT01073475

## Introduction

Globally, neonatal mortality, defined as deaths in the first 28 days of life, has dropped significantly in the last two decades, from 33 deaths per 1,000 live births in 1990 to 20 deaths per 1,000 live births in 2013 [1]. However, rates in sub-Saharan Africa and South Asia were still high in 2013, at 31 and 30 deaths per 1,000 live births respectively. Maternal mortality ratios have also declined from 380 deaths in 1990 to 210 deaths per 100,000 live births in 2013 [2]. In 2013 there were an estimated 289,000 maternal deaths, with sub-Saharan Africa accounting for 62% (179,000) and South Asia accounting for 24% (69,000) of those deaths [2].

The World Health Organization (WHO) estimates that less than 40% of all countries have an adequate civil registration system for collecting information on births and deaths and that less than half of births are registered in some developing countries, where vital registration systems are inaccurate and incomplete [2-4]. Regions with high rates of unregistered births likely have disproportionately high neonatal mortality rates and stillbirths are not recorded in many existing systems [3]. This disparity results in unreported perinatal and neonatal mortality and potentially decreases the ability to develop effective interventions to improve newborn and child survival.

Maternal and perinatal population-based registries (MPPBRs), systems to register all births, play a crucial role in understanding demographic trends in pregnancy and birth outcomes at local or regional levels [5,6]. MPPBRs are also valuable for epidemiologic research on risk factors and causes of maternal and perinatal deaths and diseases. They serve to evaluate the effects of population level interventions aimed at reducing the burden of death and disease. The ability of MPPBRs to accurately carry out these activities is highly dependent upon the accuracy and consistency of the data collected as well as the ability to make results available on a timely basis to those who can use them.

Results of several high quality perinatal registries in middle and high income countries, such as Norway and Russia, have been previously reported in the literature [7-9]. However there are few high quality MPPBRs in developing countries often due to weak healthcare systems and limited resources to ensure all pregnancies and births are accurately captured [6]. Even where population registries exist in developing countries, unless efforts are made to continuously monitor data quality, the quality of data collected may be inaccurate and unreliable [6]. Metrics that are commonly used to evaluate the quality of population-based registries include the relevance, completeness, timeliness, accuracy/validity and comparability of data obtained [10-13]. However, to date, few papers have described the monitoring efforts required to ensure that MPPBRs generate high quality data.

The Global Network for Women’s and Children’s Health Research (GN)’s Maternal Newborn Health Registry (MNHR) is one such MPPBR that provides a valuable resource to global and local knowledge of pregnancy outcomes in low and middle-income countries. The primary purpose of the MNHR is to quantify and analyze trends in pregnancy outcomes in defined low-resource geographic areas over time in order to provide population-based statistics on key pregnancy outcomes. The process of monitoring and evaluating data quality of MPPBRs may occur at multiple levels from the development and testing of data collection instruments, to proper training and oversight of data collectors, to review and verification of data collected using data metrics. This paper aims to describe the monitoring processes and metrics used to assess performance of data collection for the MNHR and illustrate how those performance metrics are used to identify potential data quality issues and evaluate the ongoing performance of the registry.

## Materials and methods

The MNHR is a prospective, population-based observational study funded by the *Eunice Kennedy Shriver* National Institute of Child Health and Human Development (NICHD) [6]. Key study variables include stillbirths, early and 28-day neonatal mortality, maternal mortality, rates of pre-eclampsia/ eclampsia, obstructed labor, hemorrhage, and infection. Additional variables obtained include antenatal care and delivery care, including Cesarean section, and neonatal resuscitation [6].

Study sites included in the MNHR include Argentina, Zambia, Guatemala, India (Belgaum and Nagpur), Pakistan, and Kenya. Additional details about the MNHR are described elsewhere [14]. The MNHR comprises approximately 100 study clusters, ranging from 10 – 24 clusters per site. Each cluster is a defined geographic region, usually based around catchment region for a health center, and has approximately 300-500 births per year.

The MNHR is overseen by a subcommittee comprised of MNHR investigators at each site. Additionally, each site employs a study coordinator and supervisors to oversee field activities. Finally, each site employs registry administrators (RAs) who oversee the data collection at each study cluster.

### Ethical approvals

The appropriate Institutional Review Boards and Ethics Research Committees of the participating institutions and the Ministries of Health of the respective countries approved the MNHR. Prior to initiation of the study, approval was sought from the participating communities through sensitization meetings. Individual informed consent for study participation is requested from each study participant. No monetary reimbursements are provided to study participants nor to the communities participating in the study. A Data Monitoring Committee, appointed by the NICHD, oversees and reviews the study at annual meetings.

### Enrollment and consent

First, to identify each pregnancy within a cluster, RAs with community health workers (CHWs) conduct community household surveys, track women of reproductive age and/or visit antenatal care clinics to screen and enroll women. Once a pregnant woman is identified as a resident of the cluster, she is asked to consent to participate and is regularly monitored for occurrence of key events during pregnancy.

### Follow-up

Using the estimated date of delivery (EDD), CHWs and RAs determine expected dates for follow-up visits to capture the outcome of the pregnancy up to 6 weeks postpartum. At delivery, key variables are recorded on data collection forms which are then reviewed and entered into the data management system (DMS) at site specific data center(s). Because deliveries documented within the MNHR occur in diverse settings ranging from family or birth attendant homes, to primary health centers, to district or tertiary care hospitals, the RAs use multiple sources of information to complete the forms. Sources of information include interviews of mother, family member present at the time of delivery, or birth attendant; medical records; and actual measurements. Information available in the medical records is used if it is determined reliable, but for some key variables such as birth weight, the RAs are encouraged to record the birth weight using instruments provided for the study rather than capturing the birth weight recorded on medical records, as instrumentation in many primary health centers is not well maintained. For each data element, the RA is given responsibility of selecting the most reliable data source.

### Data entry and management

Prior to data entry, data collection forms are manually checked for errors and missing information by supervisors and data entry staff and returned to each RA to review as needed to correct errors or ambiguities. Data entry personnel enter data forms into a data management system (DMS). Double data entry (re-keying) is performed monthly for at least 5% of the data forms per cluster using a random list of study IDs and data forms provided by statisticians at the Data Coordinating Center (DCC) to ensure consistency of data entered. After receiving lists of data edits from the DCC, the RAs make corrections on the data forms and the data entry and data management staff update the records in the site-based DMS as needed.

The DCC at RTI International (RTI) develops data collection forms and designs, maintains, and updates the data management system (DMS) centrally and at the site-level. The DMS has built-in range and skip checks to prevent errors as data are entered from paper forms into the DMS. RTI also develops detailed summary site-level and cluster-level monthly monitoring reports, which include frequencies of several variables collected at the cluster level. Monthly monitoring reports are accompanied by edit reports which flag data errors, out-of-range and inappropriate data, and missing data that were either not checked during data entry into the DMS, or that the DMS did not have the sensitivity to check. Edit reports also check for inconsistency across data forms as well as flag study IDs where delivery or follow-up information was expected but has not yet been entered.

On a monthly basis, each GN site reviews the monthly cluster-level monitoring reports with field staff to identify potential quality issues, determine the potential causes for any issues identified and develop plans for addressing those issues. On a routine basis (monthly during the early years and quarterly currently), the DCC generates a summary of potential issues based on a comparison cluster-level results to against pre-defined performance metrics (described in the section below) and reviews those reports on conference calls with the sites. These calls focus on major quality issues and development of plans at either the cluster or site level to address major performance issues.

### Performance metrics

The MNHR uses a set of metrics to provide feedback to assess the data quality. These indicators are used to monitor unexpected changes in trends that may signal poor performance within the site or technical issues related to data collection, reporting and transmission. We developed performance metrics with quantitative indicators to monitor the quality of data collected in the MNHR (Table 1).

**Table 1:** Data quality monitoring indicators used by the Global Networks’ Maternal Newborn Health Registry

Indicators	Acceptable value
Enrollment metrics	

Proportion of enrolled subjects with a consent rate obtained	>95%

Month-to-month enrollment variability factor during the past 6 months (month with highest number enrolled divided by month with lowest number enrolled)	<2.0

Pregnancy outcome metrics	

Proportion of expected delivery outcomes obtained	>95%

Proportion of deliveries with a 6-week neonatal outcome obtained.	>95%

Range of gender ratios	.80-1.30

Month-to-month birth variability factor in the past 6 months (month with highest number of births divided by month with lowest number of births)	<2.0

Ratio of expected deliveries to average monthly deliveries in the next 6 months	>70%

Minimum number of deliveries per month per cluster	25

Mortality outcome metrics	

Ratio of stillbirths to early neonatal deaths	.50-2.0

Ratio of early neonatal deaths to 6-week neonatal deaths	.60-.90

Gestational age and actual or estimated birth weight accurately recorded for miscarriages.	<20 wks <500 g

Key variable metrics	

Proportion of deliveries with a *measured* birth weight:	

Live births	>95%

Neonatal deaths	>90%

Stillbirths	>75%

Proportion that have *any* birth weight recorded:	

Live births	>99%

Neonatal deaths	>95%

Stillbirths	>95%

Proportion of enrolled subjects with maternal height, weight and timing recorded	>95%

Proportion of deliveries with delivery attendant, delivery location, delivery mode, bag and mask use, and gestational age recorded	>99%

Proportion of deliveries with birth weight collected within 7 days of birth	>95%

Process metrics	

Time between birth and measured (or estimated) birth weight recorded.	<7 days

Time between enrollment and data entry should be less than 6 weeks.	>90%

Time between enrollment date and estimated delivery date should be greater than 4 weeks.	>70%

Time between collection of delivery information and data entry should be less than 6 weeks.	>90%

Time between delivery and completion of delivery form should be less than 4 weeks.	>90%

Time between completion of delivery form and data entry should be less than 6 weeks.	>90%

Time between delivery and completion of follow-up form should be between 5 and 9 weeks.	>80%

Time between completion of follow-up form and data entry should be less than 6 weeks.	>90%

Proportion of critical edits addressed.	>80%

## Enrollment metrics

The MNHR aims to enroll pregnant women by 20 weeks gestation, although occasionally women are enrolled later or even at the time of delivery. Monitoring the time between enrollment and delivery provides an indication of whether women are being enrolled prior to delivery. Early enrollment contributes to more accurate estimation of gestational age and EDD, and ensures that important pregnancy outcomes are recorded, including stillbirths. Although the performance of this metric is dependent on whether the mother knows or reveals her pregnancy, the MNHR monitors this by estimating the number of future deliveries based on the EDDs of currently enrolled subjects compared to number of expected deliveries based on previous year total deliveries. Month-to-month enrollment variability is monitored to ensure pregnancies are captured consistently across time. High consent rates are important to ensure coverage so the study team also monitors for proper recording of consents and overall consent rates.

## Pregnancy outcome metrics

Collection of complete and accurate pregnancy outcomes is the cornerstone of the MNHR. Expected delivery rates, month-to-month variability in deliveries, and gender ratios help determine whether expected number of births is recorded in the clusters.

## Mortality outcome metrics

To monitor the accuracy and completeness of mortality data in the MNHR a number of strategies are employed. RAs have identified all local traditional birth attendants in their catchment areas and meet routinely to review birth and death records. In addition, RAs review health facility records to verify that maternal and neonatal deaths are recorded accurately. Observation of monthly mortality data for outliers, such as unusual spikes or drops in number of deaths, can help identify problems in data collection. Metrics that compare the ratio of stillbirths to early neonatal deaths and early neonatal deaths to 6-week neonatal deaths help monitor for potentially misclassified deaths.

## Key variable metrics

Variables critical to analyzing factors associated with maternal and neonatal outcomes are monitored to reduce missing data. Key variables are often associated with the collection of outcome data for ancillary studies. Collection of birth weight is an example of a key variable that has been monitored over time. The ideal is to obtain birth weight measured on a scale within 7 days of birth. However, if measured birth weight is not possible, as an alternate, RAs have been trained to obtain an estimated birth weight, using pre-defined categories of birth weight.

## Process metrics

Monitoring data collection processes, such as time between a sentinel event (e.g. delivery) and data collection time or time between data collection and data entry into the data management system, is important to understanding how efficient sites are at collecting, recording and entering data. Limiting delays in these times is considered critical because data accuracy can be compromised if data collection is distal from the time of the event. Also, data that are quickly collected and entered, can be analyzed for potential errors, and corrected in a timely manner.

## Results

Between 2010 and 2013, the seven sites in the MNHR recorded 283,496 deliveries from enrolled pregnant women. As presented in Table 1, numerous indicators are analyzed routinely to assess quality and performance of data collection in the MNHR. For the purposes of this paper, we selected 1-2 key indicators from each performance metric category, with the exception of the enrollment category, to provide illustrative examples of how data quality is monitored over time. The selected indicators were also chosen because they monitor key outcomes of the study, such as accurate and timely collection of delivery information and mortality rates.

**Figure 1:**
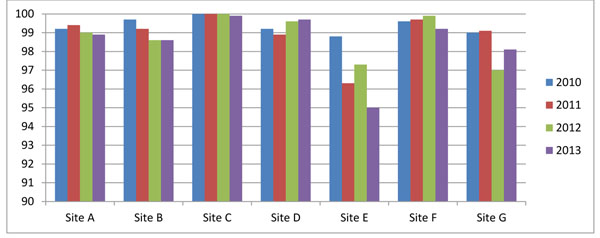
**Pregnancy outcome metrics:** Proportion of enrolled subjects with a delivery outcome (% of expected) in the Global Network’s Maternal Newborn Health Registry by year 2010-2013

Figure 1 shows that all sites have collected delivery outcomes on at least 95% of women who were expected to deliver, with the majority reaching rates up to 99%. Rates tend to fluctuate from year to year but sites are constantly working to improve follow-up of patients. Sites are provided similar results in cluster-level monitoring reports that use six-month moving averages so they can pinpoint exact clusters that are experiencing problems. Using the example of Site E in Figure 1, several clusters were identified early in 2013 with relatively low levels of follow-up. When the site investigator followed up with those clusters, we determined that the reduction in data collection was due to a large population displacement as a result of flooding. While a number of individuals were still lost to follow-up as a consequence of the displacement, the site was able to focus more resources on these clusters during the disruption period to minimize the loss.

**Figure 2:**
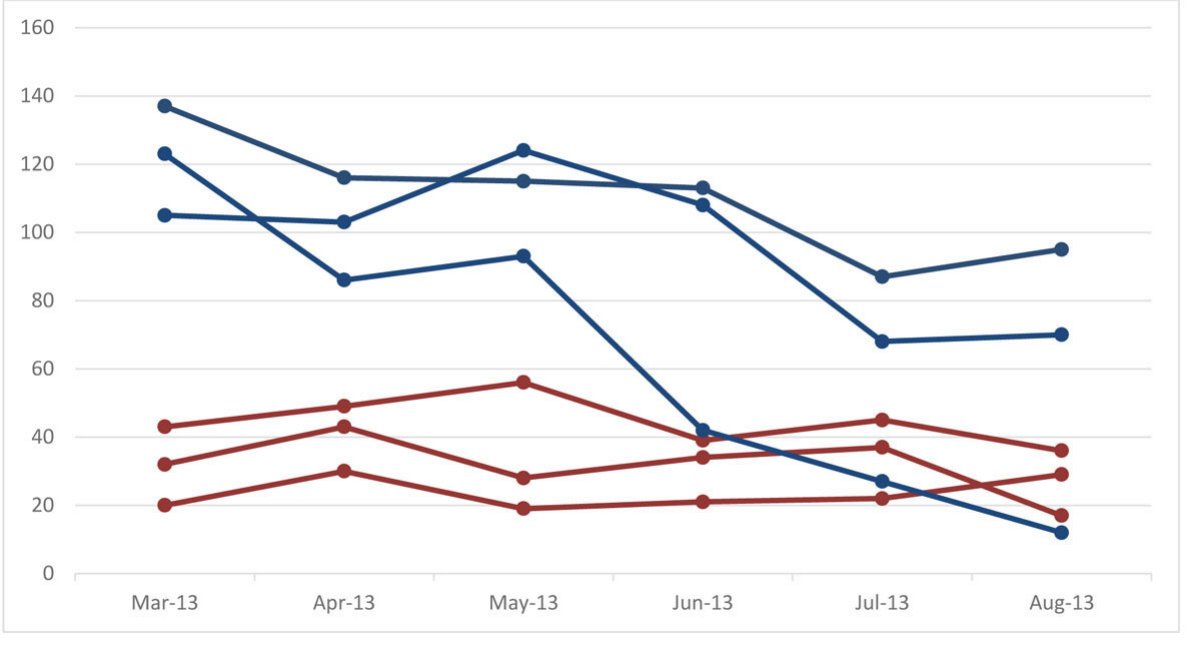
**Pregnancy outcome metrics:** Month-to-month birth variability over 6-months at a given site in the Global Network from March to August 2013 – *Blue lines represent clusters with high variability and red lines represent clusters with low variability.*

Figure 2 illustrates examples of month-to-month birth variability graphics by 6-month period and cluster. Red cluster lines demonstrate a steady horizontal trend, suggesting that births are being consistently recorded in that cluster. Blue cluster lines show a staggered downward trend over the 6-month period, suggesting that some births may be missed or not collected in a timely manner. Sites experience some seasonal variation in births, due to marriage customs, agricultural seasons and climate changes. If variability remains high over time, despite consistent efforts to capture all pregnancies, sites may consider adjusting the size or catchment area of clusters. With population movements, natural disasters, and migrations, this is often necessary. The graphics have also been used to identify staff performance issues or sites with inadequate staffing, allowing sites to adjust staffing and bring clusters into equilibrium.

**Figure 3:**
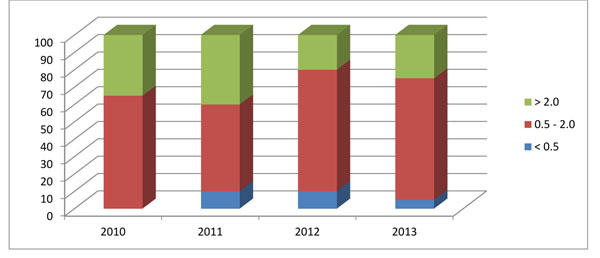
**Mortality outcome metrics:** Proportion of clusters with a stillbirth to early neonatal death ratio in the following categories at a given Global Network site by year 2010-2013.

Figure 3 illustrates an example of how stillbirths to early neonatal deaths ratios by cluster, which fall in the expected range of .50-2.0, have increased over time. The proportion of clusters with mortality ratios greater than 2.0 indicate that the number of stillbirths remains significantly higher than early neonatal deaths in certain clusters and this may be due to misclassification of deaths, or actual high number of stillbirths. In either case, sites are encouraged to investigate these types of inconsistencies to improve data quality over time.

**Figure 4:**
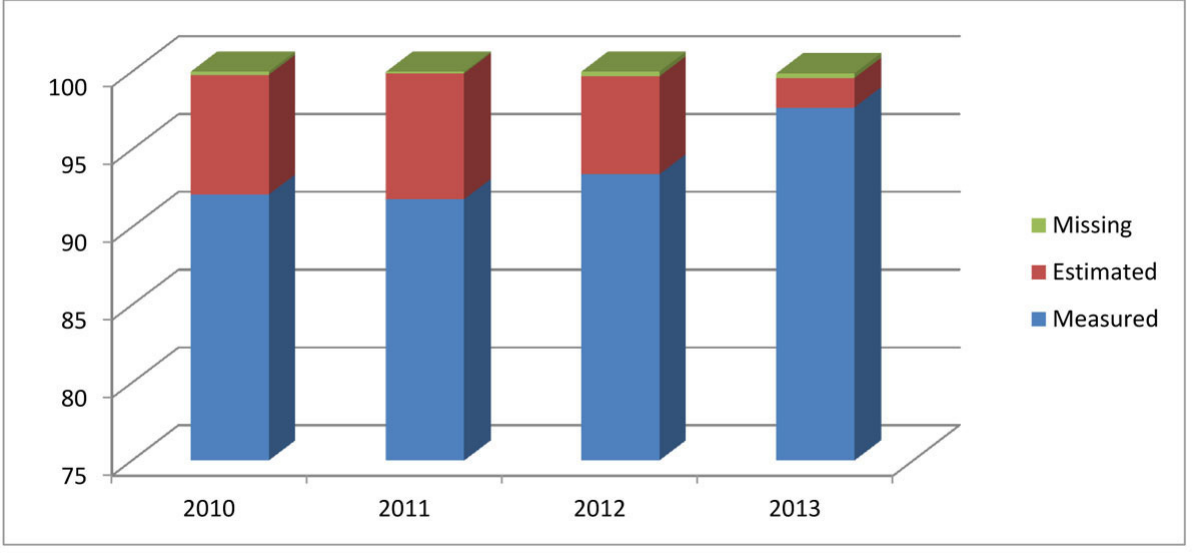
**Key variable metrics:** Proportion of missing, estimated, and measured birth weights obtained at a given Global Network site by year 2010-2013.

Figure 4 shows how the collection of a key variable, measured birth weight, improved over time in a given site. Sites aims for obtaining a measured birth weight from at least 95% of deliveries, with estimated birth weight as a second option if measured birth weight is not available. Due to the emphasis on collecting birth weight as a key outcome utilized in several studies, the ability of sites to collect this variable accurately has improved substantially over the duration of the registry.

**Figure 5:**
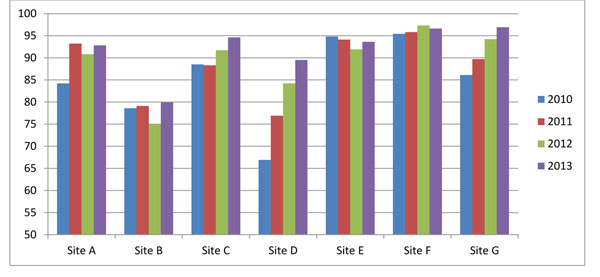
**Process metrics:** Proportion of deliveries at Global Network sites from 2010-2013 where time between delivery and completion of delivery form is <4 weeks.

Figure 5 shows how the proportion of deliveries where time between delivery and completion of delivery form was less than 4 weeks has improved over time. Sites aim to obtain delivery information as soon after delivery as possible, or within 4 weeks, so that information obtained is as accurate as possible.

## Discussion

The MNHR provides population-based indicators and trends over time of pregnancy outcomes and associated risk factors in settings where this type of information would normally be lacking or incomplete. Robust quality monitoring activities at the cluster, site, and DCC level ensure high quality data is obtained throughout the data collection and data entry process. Results of data quality metrics over time are used to monitor performance and measure consistency in data collection within sites and clusters. Furthermore, as the registry evolves, these quality control metrics continue to evolve to address specific quality issues that are identified that limit the usefulness of the data.

The illustrative results of selected indicators show that data quality monitoring efforts over time have yielded overall improvements in data collection performance of key study outcomes. Although a downward trend in the ability of some sites to obtain all delivery outcomes of enrolled subjects is observed, overall sites obtained the goal of at least 95% of delivery outcomes, with some as high as 100%. The month-to-month birth variability by cluster metric shows that whereas some clusters succeed in obtaining a consistent numbers of deliveries over time, other cluster performance is too variable, indicating that there may be obstacles to enrolling all pregnant women or obtaining all delivery outcomes in certain clusters. The increase in measured birth weight over time is an indication that monitoring this variable has proven to be an effective way to inform sites of where improvements can be made. Finally, the number of sites which were increasingly able to collect delivery information within the ideal 4 weeks’ time from delivery, shows that sites improved data collection procedures to obtain more timely, and likely more accurate, results of pregnancy outcomes.

The MNHR provides a critical foundation to the Global Network, by identifying trends in maternal and perinatal outcomes that form the basis for the design of cluster and individually randomized trials, pre-post studies, programmatic interventions, and eventually, with the goal of influencing health care practice and policy. Data from the MNHR have been used to capture outcomes for several past GN common protocols, including the Emergency Obstetric and Newborn Care (EmONC) trial [15], Antenatal Corticosteroids Trial (ACT) [16] and the Helping Babies Breath (HBB) trial [17]. Several sites have used the MNHR as a platform for data collection for sub-studies, such as the Household Air Pollution (HAP) survey and Contraception survey [18], and thus have relied on the ability of the registry to collect accurate data and follow pregnant women closely.

The high quality of data obtained by the MNHR is critical as it fills a gap that exists in civil registration systems in these low-income settings. Even where civil registration systems exist, the quality of cause of mortality data is known to be poor [19]. The processes used in implementing and monitoring the MNHR can be adopted by Ministries of Health to establish similar pregnancy tracking systems or strengthen existing systems such as the Indian Mother and Child Tracking System (MCTS). Data obtained from the MNHR permits assessment of maternal and newborn care practices in specific communities and facilities and therefore helps local health officials and policy makers plan interventions to address morbidity and mortality unique to those areas.

The MNHR also serves a critical role in contributing to global knowledge of the burden of maternal and newborn morbidity and mortality in low-income countries in general and in the specific Global Network countries. In recent years the World Health Organization (WHO) has taken the lead in assessing the quality of vital registration systems worldwide in an effort to help strengthen them [20]. At the end of 2003, coverage of death registration was less than 10% in the African region and less than 50% in the South Asia region [20].

Although monitoring mortality ratios may not necessarily indicate if deaths are being missed, it does help identify potentially misclassified deaths or an unexpected number of certain deaths. The MNHR uses mortality ratio acceptable ranges that are derived from input from the GN scientific advisory committee and validated in the literature [4,21-23]. Hill & Choi found that accurate ratios of early neonatal deaths to late neonatal deaths were unlikely to fall outside of the range of 0.66-1.26, yet in 40% of the surveys conducted, the index exceeded 2.5 [4].

Monitoring data collection processes for timeliness ensures that data are collected and transmitted regularly and provided to the data users as a feedback loop to improve the quality of data collected [13,24]. In the case of the MNHR, the time between birth and retrieval of accurate maternal and perinatal data is likely to be inversely correlated. As more time passes between the birth and the collection of its data, missing or inaccurate data (e.g. birth weight, offspring status at birth, gestational age, etc.) will be more likely. Timely collection of data also is also important for the dissemination of information for publication and time-sensitive health policy decision making.

One of the major limitations of the current quality control program is a process for evaluating the validity of the data collection forms against source documentation. While a registry would ideally involve an audit of some fraction of the data in the final registry against valid source documents, as one would typically find in a clinical trial or registry in developed countries, the diversity of data sources and the limitations of the quality and documentation associated with those sources precludes such an audit in the current setting in which the MNHR operates.

A major strength of the MNHR data quality monitoring system is that data collection instruments and procedures are uniform across all sites and monitoring metrics are developed at the DCC level. This system has been consistently applied and successfully in place across all sites for nearly 6 years. The lack of comparable systems at the GN sites limit our ability to evaluate the quality of data collected using established data quality indicators as described in studies of high income country health registries [13]. Completeness and accuracy of data collected, or the ability to ensure that no cases are missing and that data obtained can be validated by external sources, is reliant on robust civil registration systems, diagnostic and pathological reports, and death certificates, which are not often available in low-income health settings [11-13]. However, many sites utilize household surveys and hospital registers to the best of their ability to ensure that all pregnancy outcomes are captured. The completeness of enrollment data (i.e. number of consented pregnant women) is measured against historical data regarding the number of expected pregnancies occurring in the last 6 months within that cluster. In the MNHR’s predefined geographic clusters, it is reasonable to assume that enrollment and delivery rates will not significantly vary over time, although some known seasonal variation is expected and accounted for in some areas. Reports from the India Sample Registration System show that birth rates collected by the MNHR were comparable to state-specific recorded rates and therefore we are confident that the registry collects close to actual rates of pregnancy and delivery in the selected sites [25].

## Conclusions

High quality MPPBRs such as the MNHR provide key information on pregnancy outcomes to local and international health officials where civil registration systems are lacking. These data are necessary for documenting demographic trends and ensuring policies and interventions address the burden of maternal and newborn morbidity. The registry also provides a platform for assessing the impact of community/population-based interventions. Ongoing data quality monitoring using structured performance metrics is critical to ensure accuracy and completeness of data.

## List of abbreviations used

GN: Global Network; MNHR: Maternal and Newborn Health Registry; MPPBR: Maternal and Perinatal Population-Based Registry; DCC: Data Coordinating Center; DMS: Data Management System.

## Competing interests

The authors declare that they have no competing interests.

## Authors’ contributions

RLG, SSG, OP, AP, FE, EC, AG, FA, PLH, EAL, NFK, KMH, CLB and WAC designed the MNH Registry and participate in ongoing study monitoring. SSG, NVH, SCM, UVR, SMD, OP, AP, FE, EC, AG, and FA carried out data collection for the MNH registry. SSG and MKT conceived of the manuscript, KBS drafted the manuscript, JLM and DDW performed the statistical analysis. All authors read and approved the final manuscript.

## Peer review

Reviewer reports for this article can be found in Additional file 1.

## Supplementary Material

Additional file 1 Click here for file

## Declarations

This article has been published as part of *Reproductive Health* Volume 12 Supplement 2, 2015: Research reports from the NICHD Global Network for Women’s and Children’s Health Research Maternal and Newborn Health Registry. The full contents of the supplement are available online at http://www.reproductive-health-journal.com/supplements/12/S2. Publication of this supplement was supported by grants from the Eunice Kennedy Shriver National Institute of Child Health and Human Development to RTI International.
