# Supplementary material for: Data quality monitoring and performance metrics of a prospective, population-based observational study of maternal and newborn health in low resource settings
Source: Reprod Health. 2015 Jun 8;12(Suppl 2):S2. doi: 10.1186/1742-4755-12-S2-S2 (PMC4464020; doi:10.1186/1742-4755-12-S2-S2)
Supplement: Additional file 1 [file 1742-4755-12-S2-S2-S1.pdf]

Referee's comments to the authors– this sheet WILL be seen by the author(s) and published with the article

Title	**The Global Network's Maternal Newborn Health Registry Data Quality Monitoring and Performance Metrics**
Author(s)	Shivaprasad S. Goudar[1], Kristen B. Stolka[2§], Marion Koso-Thomas[3], Narayan V. Honnungar[1], Shivanand C. Mastiholi[1], Umesh V. Ramadurg[1], Sangappa M Dhaded[1], Omrana Pasha[4], Archana Patel[5], Fabian Esamai[6], Elwyn Chomba[7], Ana Garces[8], Fernando Althabe[9], Waldemar A. Carlo[10], Robert L. Goldenberg[11], Patricia L. Hibberd[12], Edward A. Liechty[13], Nancy F. Krebs[14], K. Michael Hambidge[14], Janet L. Moore[1], Dennis D. Wallace[1], Carl L Bose[15].
Referee's name	Joao Paulo Souza
Date for receipt of referee's comments	April 23, 2015

When assessing the work, please consider the following points, where applicable:

1. Is the question posed by the authors new and well defined?
2. Are the methods appropriate and well described, and are sufficient details provided to replicate the work?
3. Are the data sound and well controlled?
4. Does the manuscript adhere to the relevant standards for reporting and data deposition?
5. Are the discussion and conclusions well balanced and adequately supported by the data?
6. Do the title and abstract accurately convey what has been found?
7. Is the writing acceptable?

Please make your report as constructive and detailed as possible in your comments so that authors have the opportunity to overcome any serious deficiencies that you find and please also divide your comments into the following categories:

- Major Compulsory Revisions (which the author must respond to before a decision on publication can be reached)
- Minor Essential Revisions (such as missing labels on figures, or the wrong use of a term, which the author can be trusted to correct)
- Discretionary Revisions (which are recommendations for improvement but which the author can choose to ignore)

Where possible please supply references to substantiate your comments.

When referring to the manuscript please provide specific page and paragraph citations where appropriate.

General comments:
Many thanks for the opportunity of reviewing this manuscript. I hope that the suggestions below contribute to strengthening the report of this study.

Major compulsory revisions:
My main suggestion is that the authors follow the STROBE statement to improve reporting and decide whether results are to be presented in this manuscript. If that is the case, the results section should be substantially strengthened.

Minor essential revisions:
Title and Abstract: Please indicate the study design with a commonly use term in the title and abstract. In the methods, this is described as a "prospective, population-based observational study".

Introduction: Please consider revising the specific objective of this manuscript as follows: "This paper aims to describe the monitoring processes and metrics used to assess performance of data collection for the Global Network for Women and Children's Health Research (GN)'s Maternal Newborn Health Registry (MNHR) and present trends of the performance metrics".

[illegible]

(continue on the next sheet)

Continued:
Methods: Please correct the description of the study design. It currently says “prospective, population-based observation study“ and it would be more appropriate to say read ““prospective, population-based observational study”.
The methods section should contain only methods related details and I would strongly suggest that the authors follow the STROBE statement. The purposes of the MNHR should be presented in the introduction section and not in the methods. Please remove the sentence “The primary purpose of the MNHR is to quantify and analyze trends in pregnancy outcomes in defined low-resource geographic areas over time in order to provide population-based statistics on key pregnancy outcomes, including stillbirths, early and 28-day neonatal mortality, and maternal mortality.” Sentences such as “Monitoring data quality of the MNHR at several levels throughout the data collection and data entry process helps ensure that errors are identified early and accuracy of data is verified on a timely basis” should be placed elsewhere as it is providing a justification or discussing why a certain procedure was used.
I would like to suggest that details related to funding and project management could be presented towards the end of the methods section or in the acknowledgements.
Please revise Table 1 as it is mentioned in the methods section but makes reference to Figures with results. Suggestion: do not include the reference to the Figures.
Results: The authors state that one of the objectives of this paper is to present trends of the performance metrics, but the results section presents “examples” of selected performance indicators. I understand that there are several indicators used by the MNH Registry and some sort of prioritization needs to happen. If you are only presenting selected indicators, please provide the rational for selecting them (are they the most important ones, are they primarily guiding decision making during study implementation or analysis?). An alternative is to change the specific objectives of this manuscript in order to reflect what the authors really intend to present.
Discussion: similarly to the previous sections, please structure the discussion according to the STROBE statement, particularly expanding on the limitations.

Referee's comments to the authors– this sheet WILL be seen by the author(s) and published with the article

Title	The Global Network's Maternal Newborn Health Registry Data Quality Monitoring and Performance Metrics
Author(s)	Shivaprasad S Goudar, Kristen B Stolka, Marion Koso-Thomas, Narayan V Honnungar, Shivanand C Mastiholi, Umesh V Ramadurg, Sangappa M Dhaded, Omrana Pasha, Archana Patel, Fabian Esamai, Elwyn Chomba, Ana Garces, Fernando Althabe, Waldemar A Carlo, Robert L Goldenberg, Patricia L Hibberd, Edward A Liechty, Nancy F Krebs, K Michael Hambidge, Janet L Moore, Dennis D Wallace, Carl L Bose
Referee's name	Elizabeth Thom

When assessing the work, please consider the following points, where applicable:

1. Is the question posed by the authors new and well defined?
2. Are the methods appropriate and well described, and are sufficient details provided to replicate the work?
3. Are the data sound and well controlled?
4. Does the manuscript adhere to the relevant standards for reporting and data deposition?
5. Are the discussion and conclusions well balanced and adequately supported by the data?
6. Do the title and abstract accurately convey what has been found?
7. Is the writing acceptable?

Please make your report as constructive and detailed as possible in your comments so that authors have the opportunity to overcome any serious deficiencies that you find and please also divide your comments into the following categories:

- Major Compulsory Revisions (which the author must respond to before a decision on publication can be reached)
- Minor Essential Revisions (such as missing labels on figures, or the wrong use of a term, which the author can be trusted to correct)
- Discretionary Revisions (which are recommendations for improvement but which the author can choose to ignore)

Where possible please supply references to substantiate your comments.

When referring to the manuscript please provide specific page and paragraph citations where appropriate.

General comments:
This report outlines methods used to ensure high quality data for a registry in low resource settings. The description provides an interesting insight into some the challenges faced by the group at the data management level. Performance metrics are important and not often addressed. However, the paper would be considerably enhanced if more detail and explanation were given, as well as a more in-depth analysis and discussion of what has worked and what has not worked, and possible future uses or directions for their methods.
Major compulsory revisions:
Why are all of the results more than a year out of date (only up to 2013)? If these performance measures are really used in practice to assist the centers to identify problem areas, they have to be more current than this. The authors assert that collection of measured birth weight and time to completion of the delivery form have improved significantly but can they actually show this statistically?
Minor essential revisions:
Page 1: One of the institutional attributions of the authors (Wallace) appears to be incorrect
Page 2: The email addresses list Goldenberg twice
Page 5 (Enrollment and Consent) "She is consented" should be rephrased as 'to consent' is not a transitive verb.
Throughout 'data is' should be replaced with 'data are', since data are plural

(continue on the next sheet)

Discretionary revisions:
Page 5, Enrollment and Consent:
According to a previous publication all pregnant women who are resident or delivering within a participating cluster are enrolled. If these are the eligibility criteria for the registry, this should be explained in this report also.

Page 6, Follow-up:
How are the data collection forms ‘processed’?
The data forms are ‘cross-referenced’ with medical records to ensure accuracy and completeness. Is this done for 100% of the forms or a sample? If there is a discrepancy between the data on the form and the data in the medical record, which are considered correct.

Page 6, Data entry and management
Is there no monitoring or performance metric based on the results of double data entry? Has the group ever considered a comparison of the data on the form with the data keyed into the database to ensure that double data entry is working?

Page 6, Performance Metrics:
Why is there not a performance metric related to the accuracy of the data collected from the comparison between that data forms and the medical records? It seems that this would be as important as any.
Were the metrics decided prospectively or more pragmatically, after reviewing performance?

Page 7, Enrollment Metrics:
The authors make the point that enrollment early in gestation is important. Yet there is apparently no metric that measures this so the relevance is not clear.

Page 8
The section ‘Ethical Approvals’ seems out of place and the flow would be better if the description of IRB approvals etc was dropped into the introductory part of the Materials and Methods section.

Page 8, Results:

Why are all of the results more than a year out of date? If these performance measures are really used in practice to assist the centers to identify problem areas, they have to be more current than this. Also it might be more realistic to show the data in smaller increments than one year.

Month to month enrollment variability is shown in Figure 2 but there is no explanation of actions that the coordinating center or the group as a whole might have taken to date for those clusters with high variability, and whether they have helped.

In terms of the proportion of subjects with a delivery outcome, it does not appear that there is improvement over time and at least one site, there appears to be an opposite trend. There is no comment or speculation as to what happened or what has been done to reverse that trend. Showing the data in smaller increments than one year might be more revealing.

Supplement Editor's comments:

Please provide a more explanatory tittle of the paper. Each article should be read independently of other articles of the Supplement and readers are not supposed to be familiar about the characteristics of the Global network and the sites.

Please delete the first sentence of the Discussion section: "The MNHR is one of the first maternal and perinatal population-based registries in low-middle income countries."

In references not coming from Journals or books please provide the link and the date that this link was accessed.
